# Characterization of a Subtropical Hawksbill Sea Turtle (*Eretmocheyles imbricata*) Assemblage Utilizing Shallow Water Natural and Artificial Habitats in the Florida Keys

**DOI:** 10.1371/journal.pone.0114171

**Published:** 2014-12-17

**Authors:** Jonathan C. Gorham, David R. Clark, Michael J. Bresette, Dean A. Bagley, Carrie L. Keske, Steve L. Traxler, Blair E. Witherington, Brian M. Shamblin, Campbell J. Nairn

**Affiliations:** 1 Inwater Research Group Inc., Jensen Beach, Florida, United States of America; 2 Daniel B. Warnell School of Forestry and Natural Resources, University of Georgia, Athens, Georgia, United States of America; Texas A&M University, United States of America

## Abstract

In order to provide information to better inform management decisions and direct further research, vessel-based visual transects, snorkel transects, and in-water capture techniques were used to characterize hawksbill sea turtles in the shallow marine habitats of a Marine Protected Area (MPA), the Key West National Wildlife Refuge in the Florida Keys. Hawksbills were found in hardbottom and seagrass dominated habitats throughout the Refuge, and on man-made rubble structures in the Northwest Channel near Cottrell Key. Hawksbills captured (N = 82) were exclusively juveniles and subadults with a straight standard carapace length (SSCL) ranging from 21.4 to 69.0cm with a mean of 44.1 cm (SD = 10.8). Somatic growth rates were calculated from 15 recaptured turtles with periods at large ranging from 51 to 1188 days. Mean SSCL growth rate was 7.7 cm/year (SD = 4.6). Juvenile hawksbills (<50 cm SSCL) showed a significantly higher growth rate (9.2 cm/year, SD = 4.5, N = 11) than subadult hawksbills (50–70 cm SSCL, 3.6 cm/year, SD = 0.9, N = 4). Analysis of 740 base pair mitochondrial control region sequences from 50 sampled turtles yielded 12 haplotypes. Haplotype frequencies were significantly different compared to four other Caribbean juvenile foraging aggregations, including one off the Atlantic coast of Florida. Many-to-one mixed stock analysis indicated Mexico as the primary source of juveniles in the region and also suggested that the Refuge may serve as important developmental habitat for the Cuban nesting aggregation. Serum testosterone radioimmunoassay results from 33 individuals indicated a female biased sex ratio of 3.3 females: 1 male for hawksbills in the Refuge. This assemblage of hawksbills is near the northern limit of the species range, and is one of only two such assemblages described in the waters of the continental United States. Since this assemblage resides in an MPA with intensive human use, basic information on the assemblage is vital to resource managers charged with conservation and species protection in the MPA.

## Introduction

Hawksbill sea turtles (*Eretmochelys imbricata*) are classified as critically endangered worldwide by the International Union for the Conservation of Nature (IUCN) and are listed in Appendix 1 under the Convention on the International Trade in Endangered Species (CITES). The Western Atlantic and Caribbean populations of hawksbills continue to face a variety of threats, including loss of coral reef habitats and nesting beaches, incidental capture in fisheries, and despite protections under CITES, continued directed capture, primarily for products derived from the attractive carapace [1]. A number of historical hawksbill rookeries in the Caribbean, Western Atlantic, and Gulf of Mexico have been lost, and the long term trend (current nesting levels compared to nesting data from between 20 and 100 years ago) for hawksbill nesting was down at all nesting beaches for which trend data are available (N = 25) [1]. Analyses of historical data suggest that current hawksbill populations in the Caribbean may represent less than 1% of their historic levels [2]. Given the ability of hawksbills to migrate over large areas and through multiple jurisdictions over the course of their life cycle, and the continuing threats to hawksbill populations posed by habitat loss such as the coral reef degradation associated with global climate change, the need to understand all life history stages in all portions of their range assumes particular importance.

Hawksbills are known to occur in Florida waters, particularly in the Florida Keys and Southeast Florida, although the area is near the northern limit of their range [3]. Hawksbills are the rarest of the five species of marine turtles found in Florida waters [3] and relatively little is known of their life history there. Hawksbills are known to occur in coral reef habitats in Florida, although much of the information available on their distribution has been inferred from stranding and incidental capture data [3]. Early reports mention southeast Florida and the Florida Keys as having both the greatest abundance of hawksbills in the state and the appropriate habitat for the species [4], [5]. For this reason we initiated capture efforts in the FKNMS in 2001.

An understanding of the connectivity between developmental habitats and nesting beaches for hawksbills in the Caribbean is most simply accomplished by genetic analysis. Previous analysis of several Caribbean foraging grounds indicate varying genetic contributions from rookeries along the Central American coast and the insular Caribbean [6], but the hawksbill foraging aggregations of the Florida Keys have not been characterized.

Data collected in the KWNWR provide a rare opportunity to assess demographic composition, habitat usage and genetic origin of hawksbills in Florida waters. The major objectives of this study were to: (1) Identify important marine habitats for hawksbills in the KWNWR; (2) Characterize the size class distribution of the population; (3) Determine somatic growth rates characteristic of the assemblage; (4) Determine the natal beach origin of individuals in the assemblage; and (5) Determine the population sex ratio of the assemblage. The objectives of this study are also intended to contribute to research goals identified in the U.S. recovery plan for hawksbills produced pursuant to the U.S. Endangered Species Act [7].

## Materials and Methods

**Figure 1:**
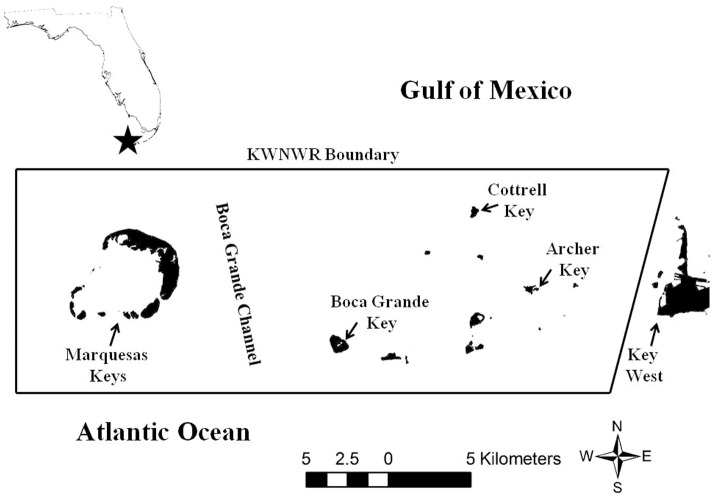
Location of the Key West National Wildlife Refuge, Monroe County, Florida.

Sampling activities were conducted in the KWNWR (Fig. 1) two to four times per year from 2001 to 2011, for a total effort of 163 days in the field. The KWNWR encompasses 521 square kilometers of open water including a portion of the United States' only continental coral reef tract. The Refuge also includes other important habitats such as mixed hardbottom/sponge areas and extensive seagrass beds. There is also an area of man-made rock rubble habitat; a derelict jetty structure known as the West Jetty, located at the edge of the Northwest Channel leading from Key West Harbor to the Gulf of Mexico. A variety of habitats within KWNWR were surveyed by vessel-based visual transects, which entailed two observers situated on an elevated platform aboard a small, shallow-draft vessel. As the vessel moved through the area, transect start and end points were defined by GPS waypoints, and the location of hawksbills sighted along the transect was recorded by GPS. Efforts were made to conduct surveys in as broad a variety of habitat types as possible within the Refuge. A total of 2563 kilometers of vessel-based transects were conducted.

### Capture Methods

Most hawksbills were captured using the rodeo method described by Ehrhart and Ogren [8]. This method entails pursuing and capturing turtles from a moving boat. Additionally, some turtles were captured by deploying snorkelers in appropriate habitats to search for and hand capture hawksbills. GPS waypoints were recorded for all hawksbill observations and those locations were plotted on available maps of benthic habitat types within the refuge using ArcView 9.3.

### Tagging and Data Collection

Once a turtle was captured, we collected morphometric data using forestry calipers and a flexible tape as described by Pritchard et al. [9]. Turtles were also weighed and photographed before release. Turtles were tagged with a National Band and Tag inconel # 681 tag applied to the proximal trailing edge of each front flipper. We also applied passive integrated transponder (PIT) tags manufactured by Destron-Fearing. These were inserted into the right front flipper at a point above the second proximal trailing scale and anterior to the first digit.

### Blood and Tissue Sample Collection

Blood samples were taken for mtDNA and sex ratio analysis. Blood was collected within five minutes following capture. We drew blood from the dorsal cervical sinus using a sterile vacutainer with no additive [10]. We collected approximately 4 ml from each turtle and added a few drops to a lysis buffer (100 mM Tris-HCL, pH 8; 100 mM EDTA, pH 8, 10 mM NaCl; 1.0% SDS) in a 1∶10 ratio. This blood was used for mtDNA haplotype analysis to determine the turtle's natal beach origin. The remaining blood was placed in a sterile vacutainer with lithium heparin and spun for ten minutes in an Adams Physician centrifuge within 8 hours of collection. Plasma was then pipetted into two 1.8 ml vials. This sample was used for testosterone radioimmunoassays to determine testosterone titer levels and gender. In the event a blood sample could not be attained, a biopsy was taken from the distal edge of one rear flipper. The small tissue sample was placed in 95% ethanol and shipped for mtDNA analysis.

### Mitochondrial DNA Analysis

DNA was extracted from blood or tissue samples from fifty individuals representing a mix of juvenile and subadult individuals using a Qiagen DNEasy kit following the manufacturer's protocols. A 740 base pair (bp) portion of the mitochondrial control region was amplified using primers LTEi9 and H950 [11]. Haplotype frequencies were compared to rookeries and other juvenile foraging aggregations from the Caribbean region from the literature for which 740 bp sequences were available (references for published haplotype data from 14 rookeries and four foraging grounds are detailed in S1 Table). Rookery contributions were estimated using a many-to-one Bayesian approach as implemented in program BAYES [12]. A many-to-many mixed stock analysis was not conducted given the small number of foraging aggregations for which 740 bp haplotypes were available and the previous demonstration of increased resolution using longer sequences [13]. Rookery contributions were weighted based on relative rookery size as measured by annual nest counts (see S1 Table for nest count references). Nest counts from the entire Mexican aggregation (rather than limiting to Holbox where samples were collected) and Doce Leguas, Cuba (rather than restricting to index beaches only) were used to prevent downward bias of rookery contributions through exclusion of proximal nesting populations.

### Serum Testosterone Analysis

Testosterone radioimmunoassays were conducted by Dave Owens at the College of Charleston and Jeffery Schwenter at the South Carolina Department of Natural Resources in Charleston, South Carolina. Individuals with testosterone levels below 261 picograms per milliliter (pg/ml) were classified as females, individuals with levels between 261 and 720 pg/ml were classified as unknown sex, and individuals with testosterone levels above 720 pg/ml were classified as males (Dave Owens, personal communication).

### Growth Rate Calculation

For individuals that were captured more than once, somatic growth rates were calculated by the division of the difference in straight standard carapace length between initial capture and subsequent recapture by days at large. For individuals with multiple recaptures, only the first and last captures were used. Individuals with a mean carapace length between first and last capture of less than 50 cm were classified as juveniles, while individuals with a mean carapace length between first and last capture between 50 and 70 cm were classified as subadults. This classification follows the criteria used by the Florida Fish and Wildlife Conservation Commission for Florida hawksbills (Meghan Koperski, personal communication). Statistical testing for significant differences (at p = 0.05) in growth rates between size classes was calculated using Student's t-test, using the Graphpad statistical program.

### Ethics Statement

This work was performed under protected species research permits from the State of Florida (Florida Fish and Wildlife Conservation Commission Marine Turtle Permit TP-125) and the National Marine Fisheries Service (permit # 16598). Field work was conducted in the waters of the Florida Keys National Marine Sanctuary and the Key West National Wildlife Refuge.

## Results

**Figure 2:**
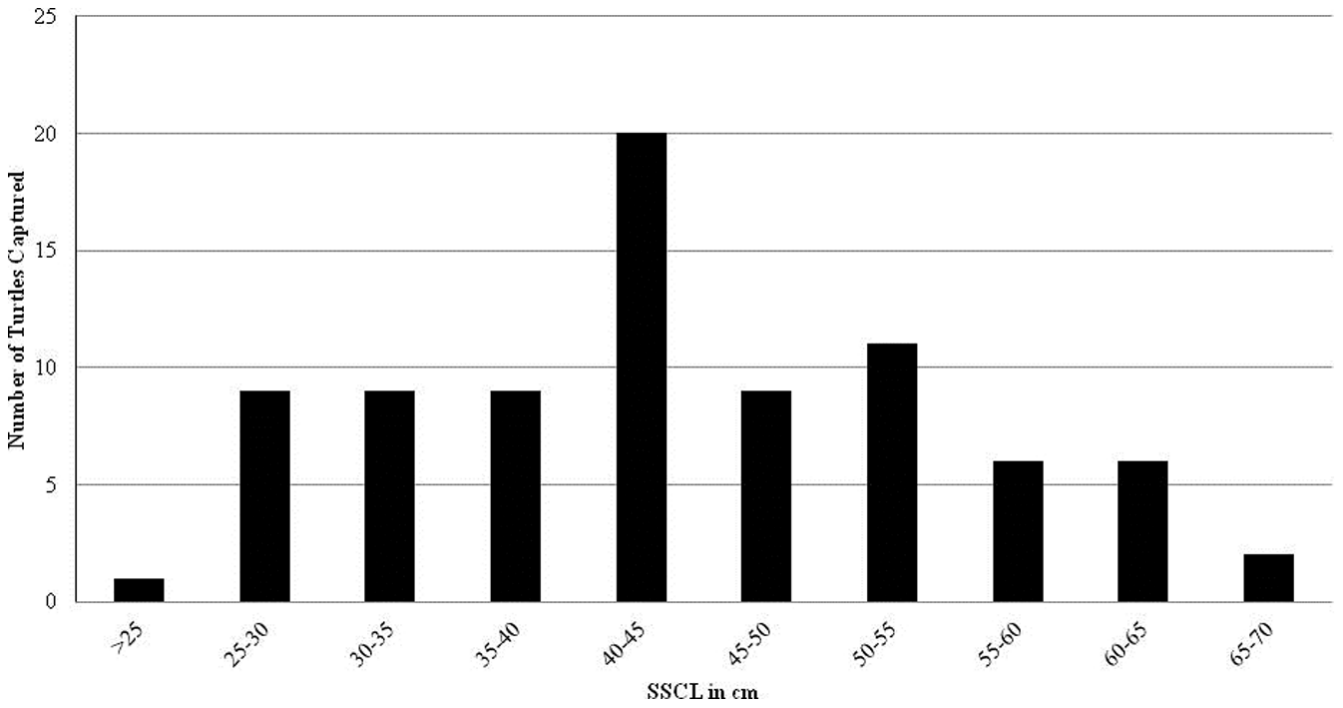
Straight standard carapace length (SSCL) size-class distribution of hawksbill turtles for all captures in the Key West National Wildlife Refuge, 2002–2011.

Straight standard carapace lengths (SSCL, measured from the nucal notch to the tip of longest pygal scute) for captured hawksbills (N = 82), ranged in size from 21.4 to 69.0 cm with a mean of 44.1 cm (SD = 10.8 cm). The size class distribution of hawksbills captured in the KWNWR is presented in Fig. 2.

**Figure 3:**
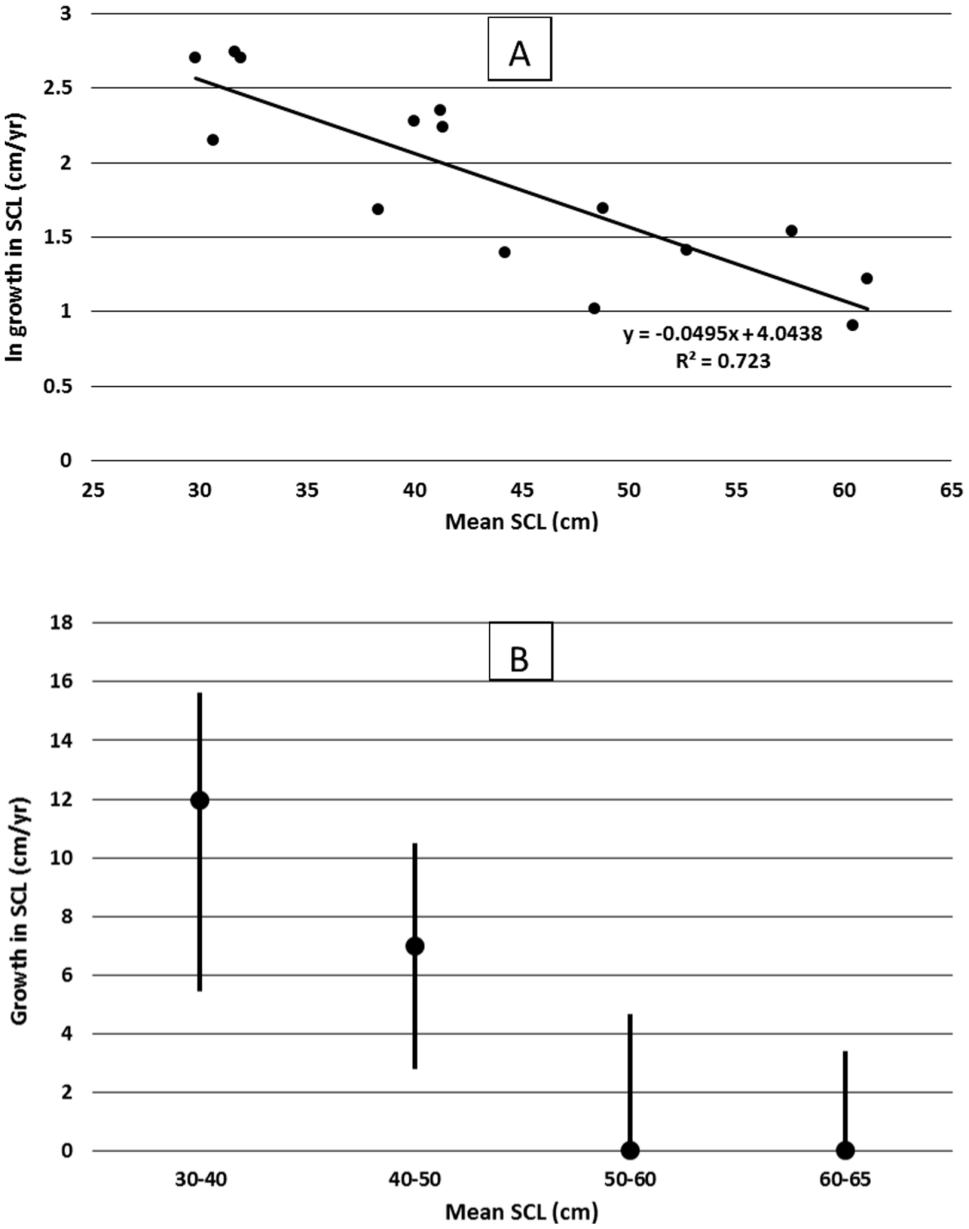
Growth in standard straight carapace length (SCL) for hawksbill turtles (n = 15) captured and recaptured within the KWNWR, 2002–2011. Plot A shows a linear fit to log transformed growth rates (R-squared  = 0.70, p<0.0001), and plot B shows mean growth rates (solid circles) and ranges (vertical lines) for hawksbills within size groupings. All sizes are means of initial and recapture SCL.

Fifteen hawksbills were captured more than once during the study and recapture intervals for these individuals ranged from 51 to 1188 days. A linear regression of log-transformed growth data (Fig. 3A) shows a highly significant (p = <0.0001) negative relationship between carapace length and growth rate. Mean growth rate and ranges for 10 cm increments in carapace length are shown in Fig. 3B. The overall mean somatic growth rate for hawksbills in the KWNWR was calculated at 7.8 cm/year (range  = 2.5 to 15.6 cm/yr, SD = 4.6 cm/yr). There was a significant difference (two tailed t-test, T = 3.844 P = 0.002) in the growth rate of individuals that were classified as subadults (SSCL 50–70 cm) (mean  = 3.7 cm/year, SD = 0.9, n = 4) and those that were classified as juveniles (SSCL<50 cm) (mean  = 9.2 cm/year, SD = 4.5, n = 11).

**Figure 4:**
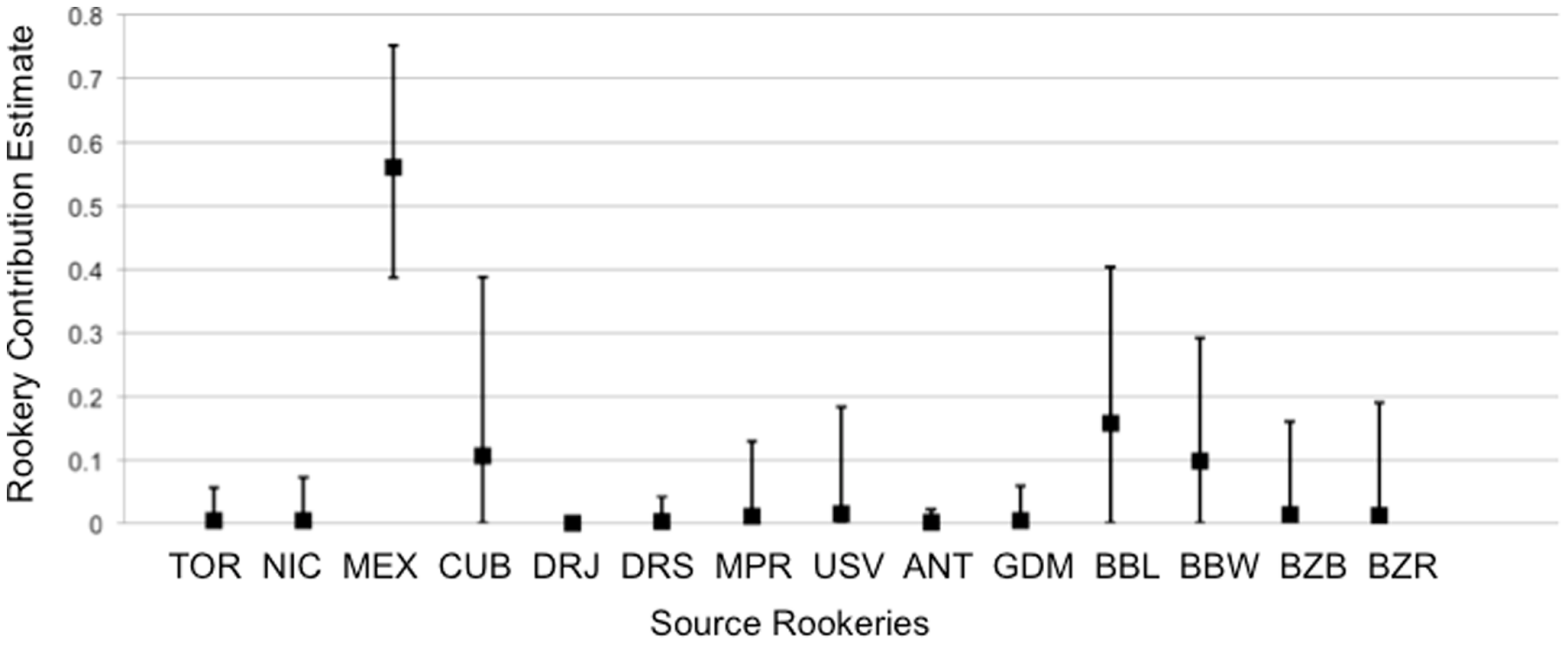
Rookery contribution estimates for juvenile hawksbill turtles foraging in the KWNWR based on mixed stock analysis weighted by rookery size. Rookery source codes are explained in S1 Table.

Fifty samples taken from juveniles and subadults for genetic analysis yielded twelve 740 bp haplotypes, including one novel haplotype that has been designated EiA89 (Genbank Accession number JX306005) (S1 Table). Besides the novel EiA89, five additional haplotypes recovered in a single individual each remain uncharacterized from rookeries but have been recorded from other foraging aggregations in the Caribbean region (S1 Table). Haplotype frequencies from the KWNWR aggregation were significantly different from all other juvenile foraging aggregations in the Caribbean with 740 bp haplotypes available, including the foraging aggregation from Palm Beach County on the Atlantic coast of Florida (Table 1). The single largest contributor to the KWNWR aggregation was the Mexican nesting aggregation accounting for approximately 56%, followed by leeward Barbados and Cuba (Fig. 4).

**Table 1:** Pairwise comparisons for 740 base pair control region haplotype frequencies for hawksbill turtle juvenile foraging aggregations in the Greater Caribbean region.

	KWGF	PBCF	CYMN	MNPR
KWGF		0.0330	0.0901	0.1310
PBCF	0.00116		0.1532	0.2363
CYMN	<0.00001	<0.00001		0.0392
MNPR	<0.00001	<0.00001	0.00008	

Pairwise FST values are above the diagonal (all values significant for alpha  = 0.01). P-values for exact tests of differentiation are below the diagonal. See S1 Table for code explanations.

An additional connection to Cuba was observed through a tag return from a hawksbill tagged in the KWNWR project area. This turtle was originally tagged on September 25, 2009 with a SCL of 60.2 cm and later recaptured 166 km away in the Varahicacos Ecological Reserve, off the northern coast of Cuba on September 19, 2012, three years later. The turtle was an incidental capture by a fisherman and no measurements were taken (Peter Eliazar, personal communication).

Testosterone radioimmunoassay data were collected from 33 individual juvenile and subadult hawksbills captured in the KWNWR. These samples contained 23 females, 7 males, and 3 individuals whose sex could not be definitively determined. The overall sex ratio of hawksbills in the KWNWR was skewed towards females, with a sex ratio of 3.3∶1.

**Figure 5:**
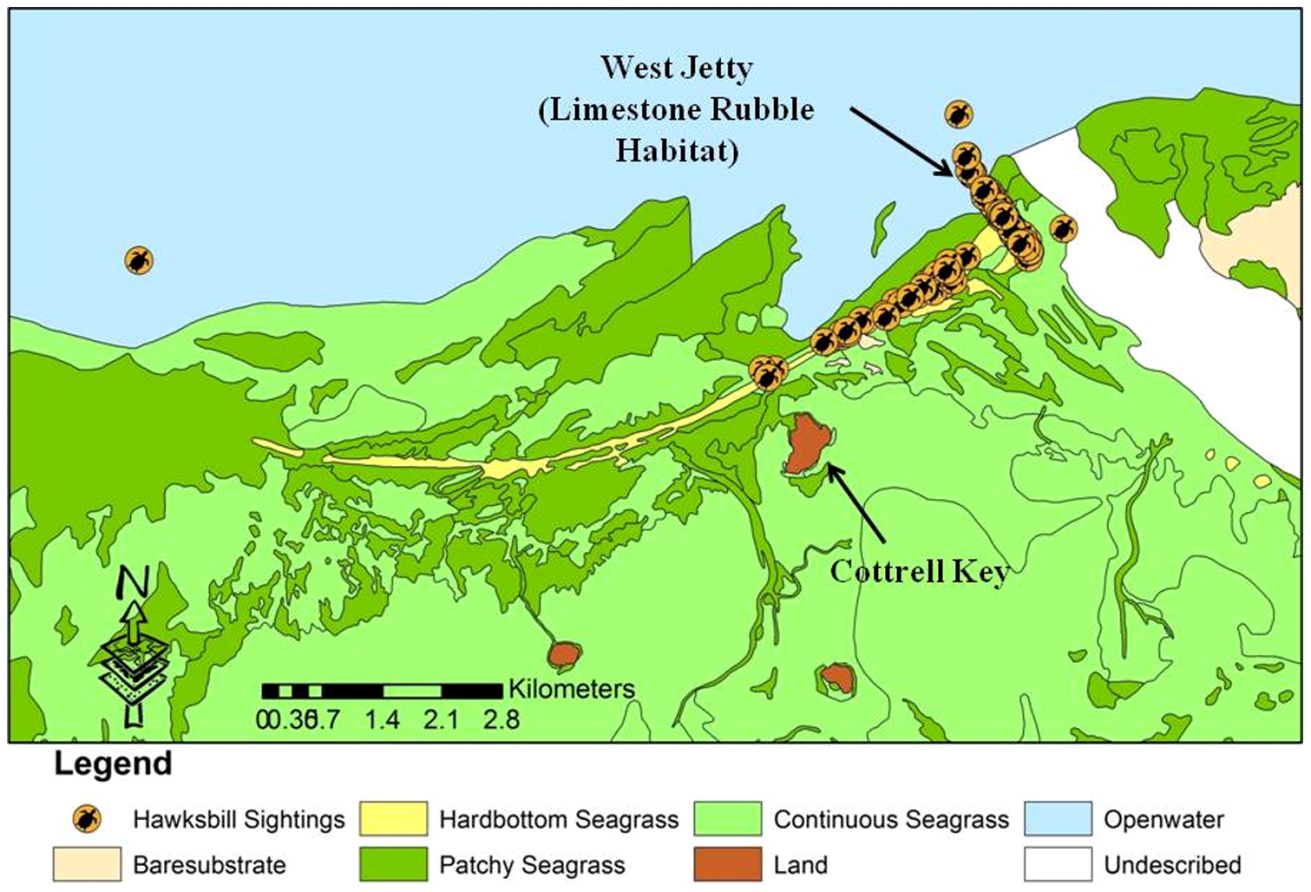
Hawksbill turtle sighting locations in the northeast KWNWR, 2001–2011.

**Figure 6:**
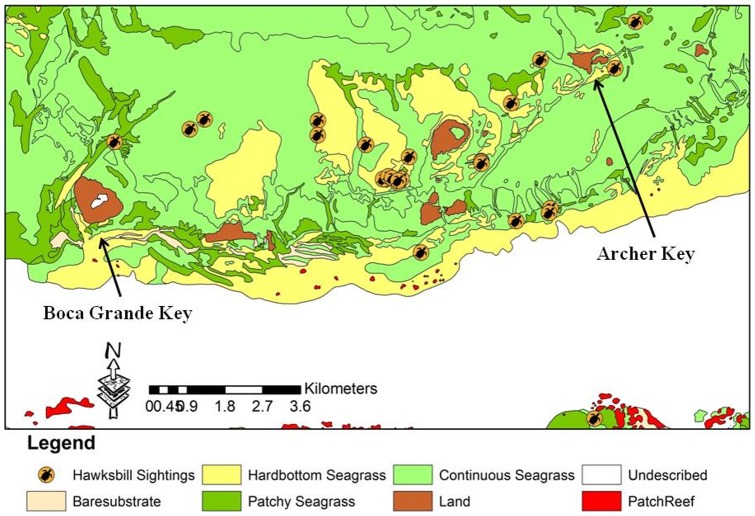
Hawksbill turtle sighting locations in the central KWNWR, 2001–2011.

**Figure 7:**
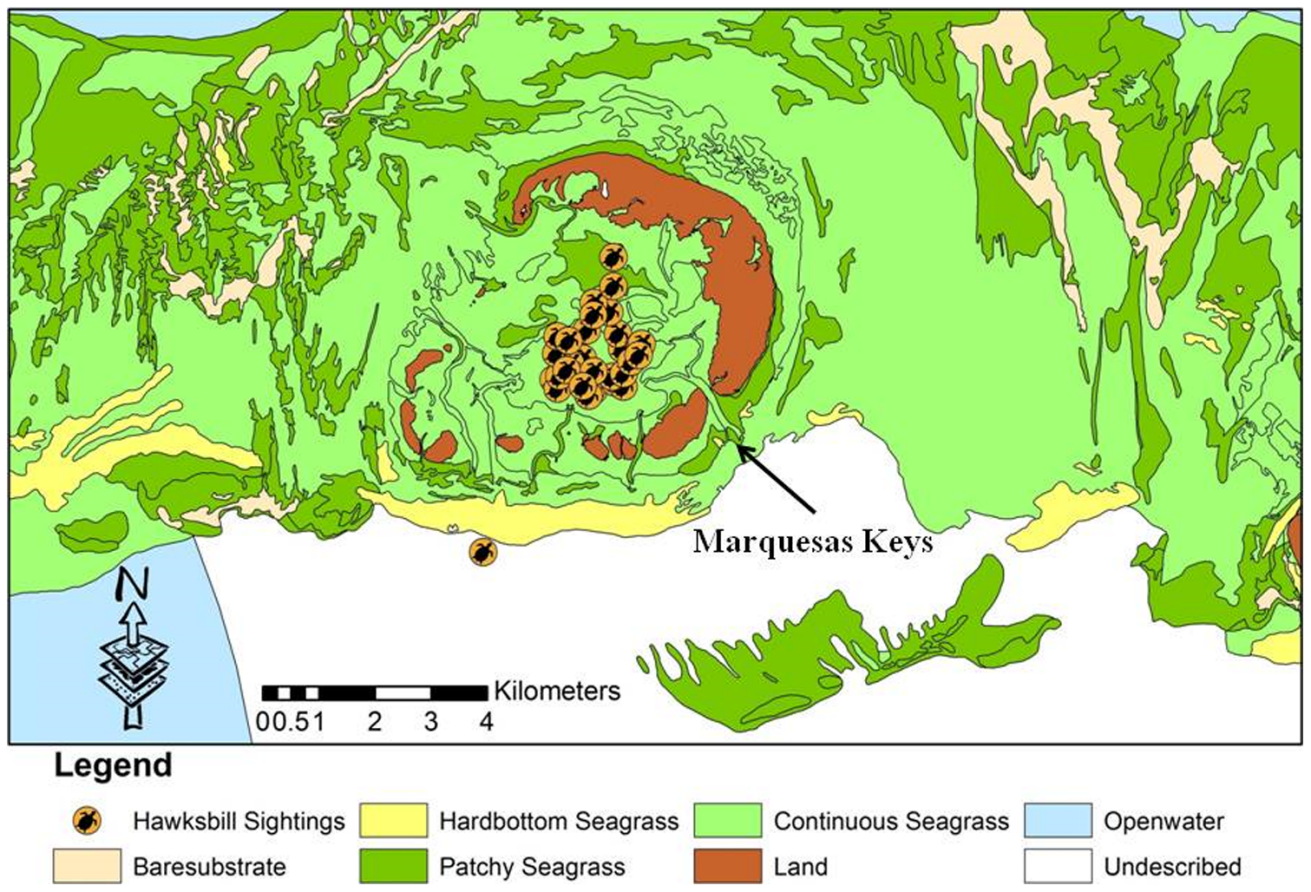
Hawksbill turtle sighting locations in the western KWNWR, 2001–2011.

Sighting data for 195 hawksbills were plotted on a map of benthic habitat types within the KWNWR. These maps were produced in 2001 by Florida Fish and Wildlife Conservation Commission staff (ocean.floridamarine.org/mrgis/description_layers_marine.htm#base). Approximately 49% of the 195 observations occurred on the limestone rubble habitat of the West Jetty. Twenty-two percent of the observations occurred over hardbottom habitat, 28% occurred over seagrass habitat, and 1% occurred over open-water habitat (Figs. 5–7). Recapture locations displayed considerable site fidelity, with the distance between initial capture and subsequent recapture locations ranging from eight to 781 meters, with a mean distance of 352 meters.

## Discussion

To properly manage the recovery of the hawksbill, information on juvenile and subadult abundance, distribution and demography is essential. This study provides data that address some of these gaps in knowledge concerning a hawksbill assemblage of particular conservation interest, one residing in a heavily used MPA and near the northern limits of its range, in an area where it has received very little study.

The hawksbills captured in the KWNWR were all juveniles or subadults, indicating that the shallow marine habitats of the KWNWR provide appropriate developmental habitat for immature hawksbills. There are relatively few studies that provide size class distributions for hawksbill populations in Florida waters [3]. One similar project to our current work is research being conducted on an offshore reef tract in Palm Beach County (PBC) in southeastern peninsular Florida. The PBC study has morphometric data from 146 individuals that shows a size class distribution mean of 56.6 cm SCL and a peak in the 51–60 cm SCL size class, with very few observations of hawksbills with a SCL of less than 40 cm [14]. In contrast, the KWNWR hawksbill assemblage has a smaller mean SCL of 44.1 cm and a peak in the size class distribution in the 40–45 cm range. Habitat type may play a role in this size class difference. The study site in PBC consists of coral reef habitat in deeper (>20 m) open ocean waters, while the hawksbill habitat in the KWNWR consists of hardbottom and mixed hardbottom/seagrass habitat in shallow (<5 m) protected waters.

It is reasonable to hypothesize that many pelagic phase hawksbills from the Caribbean Basin and Central America that are entrained in the Florida Loop Current first encounter suitable developmental habitat in the shallow reef environments of the Florida Keys. It is possible that individuals from the KWNWR assemblage migrate to the PBC study area reefs as they attain a larger size. To date we have no data demonstrating movement to the PBC study area. However, the tag return from Cuba described above does show some evidence of movement towards the Caribbean as hawksbills in the KWNWR approach maturity. This hawksbill, which was recaptured of the northern coast of Cuba, was larger than 90.2% of all hawksbills captured during this study and was considered a subadult. This tag return and the low percentage of subadult captures in the KWNWR suggest that as hawksbills approach maturity they migrate out of the shallow waters of the refuge, possibly to deeper coral reef habitats in Florida and the Caribbean. Additional evidence for movements of Florida Keys hawksbills as they attain sexual maturity is provided by Hart et al. [15]. In that study, two large subadult hawksbills (51.9 and 69.8 cm SCL) satellite tagged in the waters of Dry Tortugas National Park (less than 100 km from KWNWR) also migrated to Cuban waters. This ontogenetic shift from one developmental habitat to another may be resource dependent and has been documented in other species of sea turtles [16], [17].

Hawksbills on foraging grounds tend to show a remarkable degree of site fidelity [18], [19]. This trait allows for a greater likelihood of recapturing the same individual over time, allowing for calculations of somatic growth rates. For Atlantic and Caribbean hawksbills, Boulon [20] reported an average growth rate of 3.4 cm/yr for hawksbills ranging in size from 27.4 to 60.7 cm in the US Virgin Islands. Hart et al. [21] in a recent study at Buck Island in the US Virgin Islands, calculated a mean growth rate from 23 juvenile hawksbills of 4.1 cm/yr. In a study in the southern Bahamas, growth rates of four wild juvenile hawksbills ranged from 2.4 to 5.9 cm/yr [22]. Adult hawksbills appear to grow much more slowly, with growth rates of adult females in Costa Rica averaging only 0.3 cm/yr [23]. Growth rate data from the Palm Beach County study, based on 24 recaptures of juveniles and subadults, produced a mean growth rate of 2.9 cm/yr [14]. Growth rates were negatively correlated with carapace length, with a growth rate reduction of approximately 1 cm/yr for each 10 cm increment in SCL from 50–80 cm SCL [14]. Hawksbills in the Mona and Monito Islands of Puerto Rico also appear to have a growth rate which is negatively correlated to SCL [24].

Growth rate data from our KWNWR study is based on 15 individuals. The mean growth rate for the KWNWR hawksbills (which ranged from approximately 29–65 cm SCL) was 7.8 cm/yr. The growth rate calculated for juvenile hawksbills (those individuals with a mean at-large SCL less than 50 cm) was quite high at 9.2 cm/yr. The growth rate for the subadult hawksbills in KWNWR (those individuals with a mean at-large SCL over 50 cm) was 3.7 cm/year. Although that growth rate is based on only 4 individuals, the growth rate for subadult hawksbills in the KWNWR appears comparable with the Palm Beach County hawksbills of similar size, and is in general agreement with growth rate data for hawksbills in other regions in the Caribbean discussed above.

Genetic analysis of hawksbills in the KWNWR indicates that the refuge serves as developmental habitat for juvenile hawksbills originating from the Mexican nesting aggregation. Despite the presence of shared haplotypes between KWNWR and the PBC aggregation and a large percentage of Mexican endemic haplotypes in both foraging aggregations [14], haplotype frequencies for these two aggregations were significantly different. This suggests differential recruitment to these neritic foraging aggregations. One notable difference is the relative frequency of EiA1 in these aggregations (26% in KWNWR and 2% in PBC). Haplotype EiA1 is widespread in the western Atlantic, being the dominant haplotype in the leeward Barbados, Cuban, and Brazilian nesting aggregations [25], [26], [27]; therefore, stock contribution resolution is poor for this marker. Nonetheless, the proximity of the Cuban nesting aggregation suggests that KWNWR may serve as important nursery habitat for this severely depleted nesting population. Rookery contributions from both leeward Barbados and Cuba are consistent with particle drift modeling, which demonstrated that some proportion of hatchlings from all Greater Caribbean rookeries may pass into the Gulf of Mexico and through the Straits of Florida [28]. With the exception of most haplotypes recovered from the Mexican nesting aggregation, a few common haplotypes (EiA1, EiA9, EiA12) are widespread among Greater Caribbean rookeries, limiting inferences of demographic connectivity of rookeries as well as introducing uncertainty into rookery contribution estimates to mixed foraging aggregations. Sequencing of novel mtDNA fragments in green turtles has demonstrated rookery informative variable positions in the mitochondrial genome outside the control region [29] as well as variation in microsatellite repeat numbers in the mtDNA repetitive element [30]. Use of these techniques in hawksbill turtles may reveal cryptic structure among rookeries and improve the resolution of mixed stock analysis in the future.

Population sex ratio has important consequences for the potential recovery of sea turtle species throughout their range. Since sex is determined by incubation temperature in sea turtles, environmental variables can produce skewed sex ratios that can differ between regions. Sex ratio based on serum testosterone analysis was found to be female-biased in foraging populations of hawksbills in the US Virgin Islands [31] but were found to be near a 1∶1 ratio at Mona Island near Puerto Rico [32]. The foraging population in KWNWR appears to be strongly biased towards females, with a sex ratio of 3.3 females: 1 male. Sex ratio analysis for the Palm Beach and Broward County hawksbill assemblage also showed a sex ratio biased towards females, with a sex ratio of 2.5 females: 1 male [33].

The KWNWR contains a suite of habitat types that are utilized by hawksbills to varying extents. While hawksbills are generally thought to be closely associated with coral reefs and other hardbottom habitats, we found a surprising number (28% of observations) to be associated with seagrass dominated habitat. This observation is similar to that noted in Dry Tortugas National Park by Hart et al. [15]. Juvenile and subadult hawksbills are highly spongivorous in diet [34], [35] and we did observe that the majority of the seagrass-dominated habitats we encountered in the Refuge had numerous small hardbottom outcrops with associated sponges. We also noticed a tendency of our observations to occur near the boundaries of two habitat types, suggesting that hawksbills in the refuge may use resources from a variety of habitat types, and tend to concentrate at ecotones where those resources may be in close proximity to each other. Similar observations of hawksbills utilizing peripheral seagrass habitat have been documented in the southern Bahamas [36]. Of particular interest was the very high abundance of hawksbills noted on the West Jetty, a man-made, submerged, rock rubble structure on the edge of the Northwest Channel, which connects Key West Harbor with the Gulf of Mexico. This jetty is extensively colonized by sponges and hard and soft corals, and is located adjacent to a large area of natural hardbottom at Cottrell Key. Observations and captures along this structure represented over 53% of all hawksbill captures and over 46% of all hawksbill sightings in the refuge during this study. Similar use of artificial habitats by hawksbills has been noted on rock rubble jetty structures in Texas [37] and on deeper water artificial reefs in Palm Beach County [38]. Given the continued degradation of tropical and subtropical hardbottom and coral reef habitats due to climate change and other anthropogenic stressors, an investigation of the potential for man-made structures of this kind as particularly high value habitat for hawksbills may be warranted.

These results contribute to our understanding of hawksbill ecology in a little studied area near the northern limits of their range. Additionally, these results provide information for hawksbill conservation and management in the KWNWR.

## Supporting Information

S1 Table **Distribution of 740 base pair mitochondrial control region haplotypes for hawksbill turtle nesting populations in the western Atlantic (3 digit codes) and juvenile foraging aggregations (4 digit codes) in the Greater Caribbean region.** Data sources: A: Velez-Zuazo et al. 2008, B: LeRoux et al. 2012, C: Mortimer and Donnelly 2008, D: Revuelta et al. 2012, E: Kamel and Delcroix 2009, F: Diáz-Fernandez et al. 1999, G: Carreras et al. 2013, H: Browne et al. 2009, I: Lara-Ruiz et al. 2006, J: Vilaça et al. 2013, K: Wood et al. 2012, L: Blumenthal et al. 2009.
(XLSX)
